# The Effects of Longitudinal White Matter Hyperintensity Change on Cognitive Decline and Cortical Thinning over Three Years

**DOI:** 10.3390/jcm9082663

**Published:** 2020-08-17

**Authors:** Seung Joo Kim, Dong Kyun Lee, Young Kyoung Jang, Hyemin Jang, Si Eun Kim, Soo Hyun Cho, Jun Pyo Kim, Young Hee Jung, Eun-Joo Kim, Duk L. Na, Jong-Min Lee, Sang Won Seo, Hee Jin Kim

**Affiliations:** 1 Department of Neurology, Samsung Medical Centre, Sungkyunkwan University School of Medicine, Seoul 135-710, Korea; workcare50@gmail.com (S.J.K.); blurgroove@gmail.com (Y.K.J.); hmjang57@gmail.com (H.J.); tldmsldi@naver.com (S.E.K.); k906141h@hanmail.net (S.H.C.); torch0703@gmail.com (J.P.K.); neophilia1618@gmail.com (Y.H.J.); dukna@naver.com (D.L.N.); sangwonseo@empal.com (S.W.S.); 2 Departments of Neurology, Gyeongsang National University Changwon Hospital, Changwon 51372, Korea; 3 Department of Mental Health Research, National Centre for Mental Health, Seoul 04933, Korea; babadk2@gmail.com; 4 Neuroscience Centre, Samsung Medical Centre, Seoul 135-710, Korea; 5 Samsung Alzheimer Research Centre, Samsung Medical Centre, Seoul 135-710, Korea; 6 Departments of Neurology, Inje University College of Medicine, Haeundae Paik Hospital, Busan 48108, Korea; 7 Department of Neurology, Chonnam National University Hospital, Gwangju 501-757, Korea; 8 Department of Radiology and Imaging Sciences, Indiana University School of Medicine, Indianapolis, IN 47405, USA; 9 Department of Neurology, Myongji Hospital, Hanyang University, Goyang 10475, Korea; 10 Department of Neurology, Pusan National University Hospital, Busan 49241, Korea; eunjookim@pusan.ac.kr; 11 Department of Health Sciences and Technology, Sungkyunkwan University, Seoul 135-710, Korea; 12 Department of Biomedical Engineering, Hanyang University, Seoul 11923, Korea; ljm@hanyang.ac.kr; 13 Department of Clinical Research Design and Evaluation, Sungkyunkwan University, Seoul 135-710, Korea; 14 Department of Digital Health, SAIHST, Sungkyunkwan University, Seoul 135-710, Korea

**Keywords:** white matter hyperintensity, cortical thinning, cognitive decline, subcortical vascular mild cognitive impairment

## Abstract

White matter hyperintensity (WMH) has been recognised as a surrogate marker of small vessel disease and is associated with cognitive impairment. We investigated the dynamic change in WMH in patients with severe WMH at baseline, and the effects of longitudinal change of WMH volume on cognitive decline and cortical thinning. Eighty-seven patients with subcortical vascular mild cognitive impairment were prospectively recruited from a single referral centre. All of the patients were followed up with annual neuropsychological tests and 3T brain magnetic resonance imaging. The WMH volume was quantified using an automated method and the cortical thickness was measured using surface-based methods. Participants were classified into WMH progression and WMH regression groups based on the delta WMH volume between the baseline and the last follow-up. To investigate the effects of longitudinal change in WMH volume on cognitive decline and cortical thinning, a linear mixed effects model was used. Seventy patients showed WMH progression and 17 showed WMH regression over a three-year period. The WMH progression group showed more rapid cortical thinning in widespread regions compared with the WMH regression group. However, the rate of cognitive decline in language, visuospatial function, memory and executive function, and general cognitive function was not different between the two groups. The results of this study indicated that WMH volume changes are dynamic and WMH progression is associated with more rapid cortical thinning.

## 1. Introduction

Subcortical white matter hyperintensities (WMHs), common findings in aging, are associated with vascular risk factors, and have been recognised as a surrogate marker of cerebral small vessel disease (SVD) [1,2]. In several cross-sectional studies, WMH volume was reportedly associated with decreased cortical thickness [3,4] and cognitive dysfunction [5]. The effects of SVD change on cognitive decline have been evaluated in several longitudinal studies. The progression of SVD or WMH was shown to be associated with cognitive decline over time [6,7]. With the recent advances in imaging techniques, WM integrity can also be quantified by measuring the mobility of the water molecules in the WM microstructure. Individuals with a lower WM integrity at baseline tended to progress to cognitive impairment more often than those with a higher WM integrity in a longitudinal study [8]. In addition, a high WMH burden at baseline led to an increased risk of rapid WMH progression over time [9]. In addition, the WMH volume changes were suggested to be dynamic, indicating that WMH may progress or regress over time [10,11]. According to these studies, the change in WMH volume per year was variable. The average increased WMH volume ranged from 0.1 mL/year to 2.2 mL/year, and the average regressed WMH volume ranged from −0.02 mL/year to −0.6 mL/year [11,12,13,14,15]. Some longitudinal studies suggested that WMH regression might be due to measurement errors [11,16] or pathophysiologic changes after ischemic insult [10,17]. WMH at earlier stages may reflect altered interstitial fluid mobility and water content as “pre-visible” changes, which may be reversible; however, in later stages, demyelination and axonal damage may occur, which may be irreversible [17]. 

The effects of SVD or WMH change on cortical thickness and cognition have been simultaneously evaluated in only a few studies [18,19]. In a previous study, the SVD progression group showed more rapid cognitive decline and brain atrophy compared with the SVD stable group [19]. In another study, the WMH regression or stable group showed improved memory function and decreased brain atrophy compared with the WMH progression group [18]. However, these studies were performed among patients with varying degrees of SVD. Knowledge is limited regarding the effects of WMH change on cognitive decline or cortical thinning in patients with severe SVD at baseline, such as subcortical vascular mild cognitive impairment (svMCI). 

We hypothesised that the WMH volume in svMCI patients would show dynamic changes over time. Our previous longitudinal study showed that svMCI patients show overall cortical thinning and cognitive decline over three years [20]. We evaluated whether the patients in the WMH progression group would show more rapid cortical thinning and cognitive decline compared with patients in the WMH regression group.

## 2. Methods

### 2.1. Participants

In the present study, 87 patients with svMCI were prospectively recruited from the Memory Disorder Clinic at Samsung Medical Centre from July 2006 to August 2011, who annually underwent high-resolution 3.0-Tesla brain magnetic resonance imaging (MRI) and neuropsychological tests for three years. The patients had to meet the following criteria to be diagnosed with svMCI: (1) subjective memory complaints made by the patient or caregiver; (2) an objective cognitive impairment below −1.0 standard deviations (SD) of age- and education-matched norms in at least one cognitive domain on neuropsychological tests; (3) normal activities of daily living as determined by a clinician; (4) subcortical vascular features such as a focal neurological symptoms or signs; and (5) severe WMH on T2-weighted and fluid attenuated inversion recovery (FLAIR) images defined by periventricular WMH ≥ 10 mm and deep WMH ≥ 25 mm, which was modified from the Fazekas ischemia criteria [21]. Patients received clinical interviews and neurological examinations. All of the patients underwent a [^11^C] Pittsburgh compound B (PiB) positron emission tomography (PET) scan. Patients with territorial cerebral infarction, brain tumour, and vascular malformation were excluded based on the brain MRI. 

During the three-year follow-up period, the mean duration of follow-up was 33 months and patients repeated neuropsychological tests and brain MRI four times. After 87 patients completed the baseline neuropsychological test, 87 patients completed the first-year follow-up, 77 patients completed the second-year follow-up, and 65 patients completed the third-year follow-up. After 87 patients completed the baseline brain MRI, 87 patients completed the first-year follow-up, 77 patients completed the second-year follow-up, and 61 patients completed the third-year follow-up.

### 2.2. Acquisition of MR Images 

We acquired standardized 3D T1 turbo field echo images and 3D FLAIR images from all of the patients using the same 3.0 T MRI scanner (Philips 3.0 T Achieva; Philips Health care). Briefly, the 3D T1 MR images were set to the following parameters: sagittal slice thickness, 1.0 mm, over contiguous slices with 50% overlap; no gap; a repetition time of 9.9 ms; an echo time of 4.6 ms; a flip angle of 8°; and a matrix size of 240 × 240 pixels reconstructed to 480 × 480 over a field of view of 240 mm. The 3D FLAIR image parameters included the following: axial slice thickness of 2 mm; no gap; repetition time of 11,000 ms; echo time of 125 ms; flip angle of 90°; and matrix size of 512 × 512 pixels.

### 2.3. Measurement of Longitudinal WMH Volume 

First, the baseline WMH volume was quantified using an automated algorithm, as previously described [22]. The baseline WMH candidate regions on the FLAIR images were extracted by applying a classification method and morphological operation to 3D T1-weighted images. To extract the baseline WMH, a threshold method was applied to the FLAIR images within the WMH candidate regions. Although the threshold value was selected considering the range of image intensities, segmented results could contain false-positive or false-negative regions, depending on the extent of WMH. The rate of agreement between two neurologists was 92.3%. If the results contained an error, the threshold value was reselected through visual inspection by two raters, and they reached a consensus in the case of discrepancy.

Second, the longitudinal WMH volume change was measured by evaluating the difference between baseline and follow-up FLAIR images [23]. Each follow-up brain image was transformed separately into a baseline brain space using affine transform. Because the shooting time of each longitudinal image was different, each image intensity was converted to a z-score. Then, the longitudinal WMH volume change was measured using the difference in z-score between baseline and follow-up FLAIR images.

Finally, patients were divided into the WMH progression (baseline WMH volume < last follow-up WMH volume) and WMH regression (baseline WMH volume > last follow-up WMH volume) groups based on the WMH volume change. 

### 2.4. Acquisition of [^11^C]PiB PET and Data Analysis

To measure the amyloid burden, all of the patients with svMCI underwent [^11^C]PiB PET scans with a Discovery Ste PET/computed tomography scanner (GE Medical Systems, Milwaukee, WI, USA). [^11^C]PiB PET scanning was performed using 3D scanning mode examining 35 slices of 4.25-mm thickness, spanning the entire brain. The CT scan was performed for attenuation correction, and 60-min and 30-min emission static PET scans were initiated after the injection of a mean dose of 420 MBq [^11^C]PiB. The specific radioactivity of [^11^C]PiB for patients was >1500 Ci/mmol at the time of administration, and the radiochemical yield was >35%. All of the PET studies had >95% in the radiochemical purity of the tracer.

The PiB PET images were co-registered to individual MRIs, which were normalized to a T1 MR image template. 

To measure the PiB retention, the cerebellar grey matter was used as a reference region [24]. We selected 28 cortical volumes of interest from the left and right hemispheres using the annotated anatomical labelling (AAL) atlas. The cerebral cortical volumes of interest consisted of the bilateral frontal cortices, posterior cingulate gyri, parietal, lateral temporal, and occipital areas. The regional cerebral cortical uptake ratios were calculated by dividing each cortical volume of interest uptake ratio by the mean uptake of the cerebellar cortex (cerebellum crus 1 and crus 2). The global PiB standardised uptake value ratio (SUVR) was calculated from the volume-weighted average uptake ratio of the bilateral 28 cerebral cortical volumes of interest. Patients were defined as PiB positive if their global PiB retention ratio was more than 2.0 SD (PiB retention ratio > 1.5) from the mean of the healthy controls [25]. 

### 2.5. Cortical Thickness Analyses

The process of the cortical thickness measurement was described in previous longitudinal studies [20,26]. T1-weighted images were processed using the standard Montreal Neurological Institute anatomical pipeline. The native MRI images were registered to a standardized stereotaxic space through a linear transformation [27], and the N3 algorithm was used to correct the images for intensity-based non-uniformities caused by non-homogeneities in the magnetic field [28]. Then, the registered and corrected images underwent segmentation into white matter, grey matter, and nonbrain tissue using the Intensity-Normalized Stereotaxic Environment for Classification of Tissues algorithm [29]. The surfaces of the inner and outer cortex were automatically extracted using the Constrained Laplacian-Based Automated Segmentation with Proximities algorithm [30]. Although cortical thickness was defined as the Euclidean distance between the linked vertices of these areas [30], the values were calculated in the native brain spaces, rather than using the Talairach space, given the existing limitations in linear stereotaxic normalization. The MR volumes in the native space were transformed into the stereotaxic space with a linear transformation matrix, and the reconstructing process was performed using the inverse transformation matrix [31]. Subsequently, the thickness value was spatially normalized using surface-based two-dimensional registration with a sphere-to-sphere warping algorithm, meaning that the vertices of each subject were non-linearly registered to a standard surface template [32]. We used intracranial volume (ICV) as a covariate to control for brain volume in the statistical analyses. For the global and lobar regional analyses, the data that were previously manually categorised to lobes with a high inter-rater reliability [31] were registered to the template. Finally, the cortical surface of each subject was divided into frontal, temporal, parietal, and occipital lobes via the automated processes. 

### 2.6. Neuropsychological Tests

Patients underwent annual neuropsychological tests using the Seoul Neuropsychological Screening Battery (SNSB) [33,34]. SNSB consists of several tests for language and related functions, visuospatial functions, verbal and visual memory, and executive functions. The scores of each domain in the SNSB were considered abnormal when they were lower than −1.0 SD for age- and education-matched norms. In this study, domain composite scores were derived by summing the selective tests scores for each subdomain [35]. The language and related functions domain score were derived from the raw score of Korean-version of the Boston Naming Test (0–60). The Rey Complex Figure Test (RCFT) copy score was used for the visuospatial functions domain (0–36). The memory domain was calculated from the sum of the immediate-recall score, 20-min delayed-recall score, and a recognition score from the Seoul Verbal Leaning Test (three learning/free recall trials of 12 words, a 20-min delayed-recall trial for these 12 items, and a recognition test) and RCFT (0–168). The executive functions domain was calculated from the sum of the phonemic and semantic Controlled Oral Word Association Test and the Stroop Colour Test (colour reading of 112 items during a 2-min period; 0–173). All of the patients also performed the Clinical Dementia Rating Scale Sum of Boxes (0–18) and the Korean version of the Mini-Mental State Examination (0–30). All of the tests were performed by experienced staff and supervised by board-certified clinical neuropsychologists. 

### 2.7. Statistical Analyses

To compare the demographic variables between the WMH progression and WMH regression groups, the Student’s *t*-test was performed for continuous variables and the Chi-square test or Fisher’s exact test was performed for categorical variables. 

To evaluate the effect of the longitudinal change of the WMH volume on the longitudinal cognitive decline and cortical thinning, a linear mixed effects model was performed. We included subjects and slope as random components, and baseline age, sex, time, group, PiB PET SUVR [25], ICV (for analysis of the cortical thickness), and the interaction between groups and time as the fixed components, because these factors could affect the WMH volume. We also performed a sub-analysis after excluding the amyloid PET positive svMCI patients. For the statistical analyses, SPSS 21.0 was used (SPSS Inc., Chicago, IL, USA).

To evaluate the topography of the cortical thinning associated with a change in WMH volume, MATLAB-based toolbox was used (available online through the University of Chicago: https://galton.uchicago.edu/faculty/InMemoriam/worsley/research/surfstat). To blur each cortical thickness map, a full-width half-maximum diffusion smoothing of 20 mm was used, resulting in an increased signal-to-noise ratio and statistical power [36]. Linear mixed effects models were used vertex-by-vertex to analyse the localised differences and the statistical map of cortical thickness on the surface model. Baseline age, sex, time, group, ICV, PiB PET SUVR, and the interaction between groups and time were entered as the fixed effects, and the subject was entered as a random effect. Because multiple comparisons were performed for 81,924 vertices, an uncorrected *p*-value of < 0.01 was considered statistically significant.

### 2.8. Standard Protocol Approval, Registration, and Patient Consent

The Institutional Review Board of the Samsung Medical Centre approved the study and all of the patients signed informed consent (IRB no. 2008-08-018).

## 3. Results

### 3.1. Demographics

The demographics and clinical characteristics at baseline are summarised in Table 1. Among the 87 patients, 70 showed a progression of WMH volume and 17 showed a regression of WMH volume over a three-year period. Baseline demographics, vascular risk factors, SVD burden, and antiplatelet use were not significantly different between the WMH progression and WMH regression groups. The total WMH volume at baseline was 40.98 ± 29.13 mL in the WMH progression group and 38.97 ± 24.72 mL in the WMH regression group. In the WMH progression group, the mean WMH volume increased 0.29 mL per year (*p* < 0.001) and in the WMH regression group, WMH volume decreased 0.06 mL per year (*p* = 0.006).

### 3.2. Longitudinal Change of Cortical Thickness 

In all of the svMCI patients, the patients in the WMH progression group showed more rapid cortical thinning in the frontal, temporal, parietal, and occipital lobes than the patients in the WMH regression group (Table 2). A sub-analysis including only PiB negative svMCI patients also showed that patients in the WMH progression group had more rapid cortical thinning in the frontal, temporal, and parietal lobes than patients in the WMH regression group (Table 2). We further calculated the delta WMH for each year and ran correlation analyses with this value. However, delta WMH was not correlated with longitudinal cortical thinning (Supplementary Table S1).

**Figure 1:**
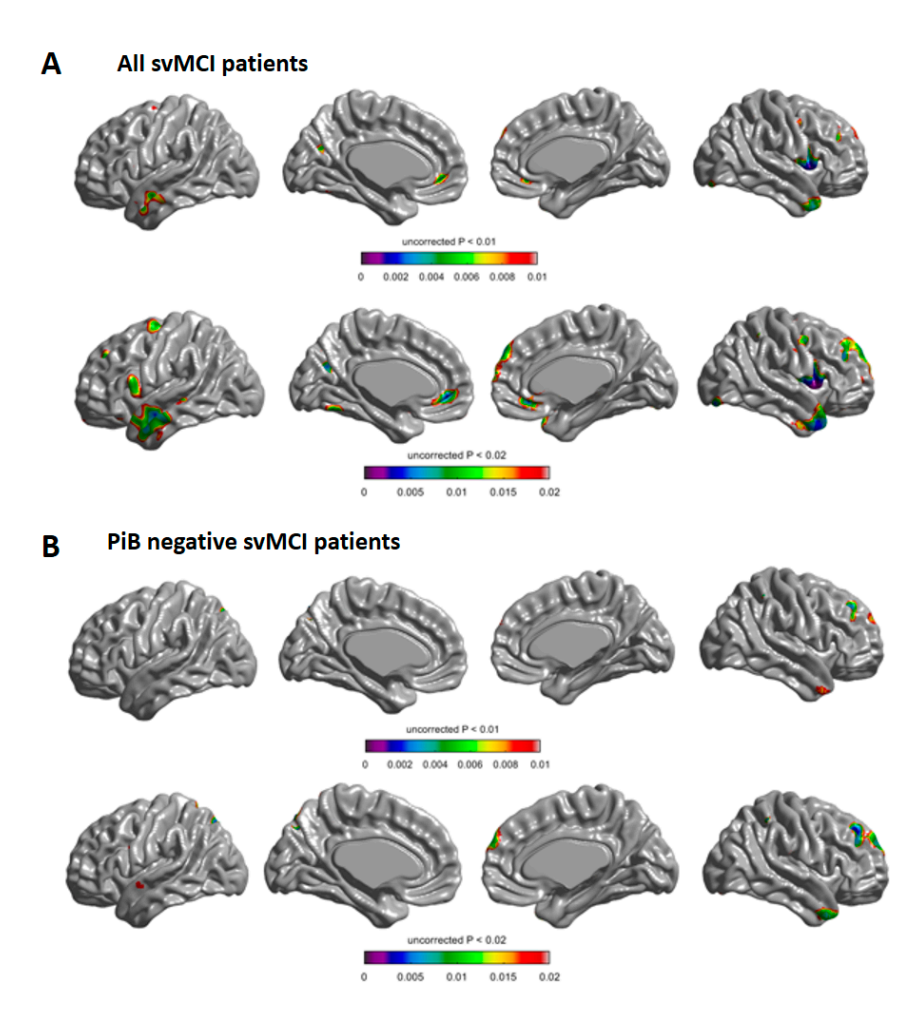
Longitudinal change of cortical thickness in the white matter hyperintensity (WMH) progression group compared with the WMH regression group in all of the subcortical vascular mild cognitive impairment (svMCI) patients (**A**) and in PiB negative svMCI patients (**B**). Compared with the WMH progression group, the WMH regression group demonstrated more rapid cortical thinning in the coloured areas. Baseline age, sex, time, group, intracranial volume, Pittsburgh compound B standardised uptake value ratio, and the interaction between the groups and time were entered as fixed effects, and subject was entered as a random effect (uncorrected *p* < 0.01 in the upper row and *p* < 0.02 in the lower row).

A statistical map showed that in all svMCI patients, the WMH progression group had more rapid cortical thinning in the bilateral frontal and lateral temporal regions than the WMH regression group (Figure 1A). A sub-analysis including only PiB negative svMCI patients also showed that the WMH progression group had more rapid cortical thinning in the right frontal and bilateral temporal regions than the WMH regression group (Figure 1B).

### 3.3. Longitudinal Change of Cognition

In all of the svMCI patients, the rates of cognitive decline in language, visuospatial function, memory, executive function, MMSE, and CDR-SB were not different between the WMH progression and WMH regression groups (Table 3). Similarly, in the PiB negative svMCI patients, the rates of cognitive decline in language, visuospatial function, memory, executive function, MMSE, and CDR-SB were not different between the WMH progression and WMH regression groups (Table 3). Further analysis showed that there was no significant difference in the rate of cognitive decline between the WMH fast progression group (*n* = 35) and the WMH slow progression group (*n* = 35).

## 4. Discussion

In the present study, dynamic changes in WMH were observed in svMCI patients over a three-year period. The major findings of the study were the following: (1) among the 87 svMCI patients, 17 patients (19.5%) showed a regression in WMH volume over a three-year period; (2) compared with the WMH regression group, the WMH progression group showed more rapid cortical thinning in the frontal and lateral temporal regions; and (3) a significant difference in the rate of cognitive decline was not observed between the WMH regression and WMH progression groups. Collectively, we suggest that the change in WMH volume was dynamic in svMCI patients, and WMH progression was associated with more rapid cortical thinning.

Our first major finding in the study was that 19.5% of svMCI patients showed significant WMH regression, while others showed WMH progression over a three-year period. This finding was in agreement with a recent report in which SVD markers demonstrated dynamic changes, as 20.3% of patients showed SVD regression over 8.7 years [19]. In another study of AD patients with varying degrees of SVD, the authors reported that the WMH volume was dynamic because it shrank, grew, or remained stable over time [14]. Despite recent published reports, the present study is unique because all of the svMCI patients in the cohort had a severe level of WMH volume at baseline, which corresponds to Fazekas III. Several hypotheses for WMH regression are possible [11,14,18]. First, the baseline WMH may have been due to transient inflammatory processes, which might resolve over time. Transient fluid-related WM damage might resolve before permanent axonal injury or demyelination [17]. Second, reversible ischemic injury might heal over time [18,19]. Third, pathophysiological changes such as blood–brain barrier disruption and the clearance of fluid after a small subcortical stroke could also be a possible underlying mechanism [14,37].

Significant differences were not observed in the demographics or baseline vascular risk factors between the WMH progression and WMH regression groups, which was in agreement with previous studies [18,19], although in one study, the body mass index in the WMH progression group was significantly higher than in the WMH regression group [19].

Second, the WMH progression group was associated with more rapid cortical thinning than the WMH regression group over a three-year period. Specifically, the WMH progression group showed rapid cortical thinning in the bilateral frontal and lateral temporal regions, which was in agreement with our previous study showing that lacunae progression was associated with cortical thinning in the frontal regions [26]. A sub-analysis, which included only amyloid negative svMCI patients, also showed that patients in the WMH progression group had more rapid cortical thinning in the frontal, temporal, and parietal lobes than patients in the WMH regression group. To date, chronic ischemia in the brain leads to demyelination and axonal loss, which results in WMH [21]. In addition, microglial and endothelial activation in the interstitial tissue of the brain leads to neuronal loss, resulting in cortical thinning [38]. For these reasons, WMH accompanies cortical thinning [39]. However, in the early phase of ischemia, interstitial fluid in the oedematous lesion may be reversed and the transient inflammatory process might heal [17,18], thereby reducing WMH and protecting against neuronal loss and cortical thinning [18,19]. Thus, these pathomechanisms might explain the dynamic change of WMH and its relationship with cortical thinning. In our additional analysis, we could not yield a significant correlation between the delta WMH for each year and cortical thinning. This might be due to a small variation in the delta WMH.

Finally, our data showed that in svMCI patients, the rate of cognitive decline was not significantly different between the WMH progression and WMH regression groups. A sub-analysis including only PiB negative svMCI patients also showed that the rate of cognitive decline was not different between the WMH progression and WMH regression groups. In contrast to our results, there are studies that have shown that the WMH progression group had a more rapid cognitive decline than the WMH regression or stable group [18,19]. The discrepancy might be explained by the different degrees of WMH burden in each study cohort. Patients in previous studies had varying degrees of WMH burden; however, the patients in this study had a severe WMH burden at baseline. Therefore, although the WMH regression group showed an overall net regression in WMH volume, severe WMH at baseline may have already led to a vicious cascade of network disruption, resulting in significant cognitive decline [19]. It is also possible that we might not have found a significant difference in the rate of cognitive decline because of the relatively small sample size.

The strength of the current study was that patients with severe WMH were prospectively recruited, and cortical thickness and cognitive function were repeatedly measured. However, the present study had several limitations. First, the sample size was relatively small. Thus, statistical differences in the rate of cognitive decline between the WMH progression and WMH regression groups, or correlation between the delta WMH for each year and cortical thinning may not have been found. The results should be interpreted with caution, and need to be confirmed in studies with a larger sample size. Second, three years of follow-up is not sufficient to observe biological changes associated with WMH dynamics, and studies with longer follow-up period are needed. Finally, whether strict control of vascular risk factors and a healthy lifestyle may mitigate WMH progression should be investigated in future studies. Despite these limitations, the results of the present study highlight the importance of dynamic WMH volume changes, which affect cortical thickness.

## 5. Conclusions

In conclusion, we found that the change in WMH volume was dynamic, as some svMCI patients showed WMH regression, while others showed WMH progression. WMH progression was associated with more rapid cortical thinning in the frontal and temporal areas. Our results suggest that identifying the dynamic change in WMH volume in svMCI patients could be valuable for predicting the rate of neurodegeneration. Further investigations are needed to assess whether strict risk factor control slows down WMH progression and protects cortical thinning or cognitive decline.

## Figures and Tables

**Table 1:** Demographics and clinical characteristics of patients in the WMH progression and WMH regression groups at baseline.

	WMH Progression (*n* = 70)	WMH Regression (*n* = 17)	*p*-Value
Age (mean ± SD), years	72.1 ± 6.5	71.8 ± 8.7	0.158
Female, *n* (%)	39 (55.7)	12 (70.6)	0.264
Education (mean ± SD), years	9.9 ± 5.5	8.1 ± 5.0	0.229
Cardiovascular risk factors, *n* (%)			
BMI (kg/m^2^)	25.05 ± 4.39	22.97 ± 3.47	0.073
Hypertension	54 (77.1)	13 (76.5)	1.000
Diabetes mellitus	15 (21.4)	5 (29.4)	0.526
Hyperlipidaemia	21 (30)	3 (17.6)	0.378
APOE4, *n* (%)	17 (24.3)	6 (35.3)	0.370
Anti-platelet agent, *n* (%)	62 (88.6)	14 (82.4)	0.443
Anti-coagulant agent, *n* (%)	1 (1.4)	0 (0.0)	0.014
Baseline SVD markers			
WMH (mean ± SD), mL	40.98 ± 29.13	38.97 ± 24.72	0.794
Microbleed (mean ± SD), *n*	5.2 ± 12.2	3.6 ± 6.3	0.648
Lacunae (mean ± SD), *n*	6.3 ± 7.3	4.3 ± 4.1	0.254
PiB PET			
PiB SUVR	1.48 ± 0.40	1.53 ± 0.38	0.711
PiB positive (SUVR > 1.5), *n* (%)	18/63 (28.6)	4/10 (40)	0.476
Cortical thickness (mean ± SD), mm	2.84 ± 0.16	2.77 ± 0.18	0.130
Cognition			
Language	40.20 ± 9.08	36.24 ± 12.22	0.223
Visuospatial function	27.07 ± 7.97	27.56 ± 7.79	0.820
Memory	75.76 ± 17.54	76.88 ± 21.60	0.845
Executive function	97.40 ± 35.74	87.88 ± 26.80	0.230
MMSE	25.91 ± 3.13	24.82 ± 3.84	0.290
CDR-SB	1.29 ± 1.07	1.74 ± 1.64	0.295
Mean follow-up duration	34.01 ± 10.13	29.06 ± 13.48	0.095

Abbreviations: WMH—white matter hyperintensity; SD—standard deviation; BMI—body mass index; SVD—small vessel disease; APOE—apolipoprotein E; PiB—Pittsburgh compound B; PET— positron emission tomography; SUVR—standardised uptake value ratio; MMSE—mini-mental status examination; CDR-SB—clinical dementia rating-sum of boxes.

**Table 2:** Longitudinal change of cortical thickness in the white matter hyperintensity (WMH) progression group compared with the WMH regression group.

	All svMCI (*n* = 87)		PiB Negative svMCI (*n* = 51)	
	B (SE)	*p-*Value	B (SE)	*p-*Value
Global thickness	−0.034 (0.012)	0.006	−0.027 (0.012)	0.030
Frontal lobe	−0.041 (0.013)	0.002	−0.028 (0.013)	0.033
Temporal lobe	−0.040 (0.016)	0.014	−0.035 (0.015)	0.024
Parietal lobe	−0.034 (0.012)	0.005	−0.032 (0.012)	0.009
Occipital lobe	−0.025 (0.011)	0.029	−0.019 (0.014)	0.173

Abbreviations: WMH— white matter hyperintensity; SE—standard error. A linear mixed effects model was performed after adjusting for baseline age, sex, time, group, intracranial volume, Pittsburgh compound B standardised uptake value ratio, and the interaction between the groups and time.

**Table 3:** Longitudinal change of cognitive function in the WMH progression group compared with the WMH regression group.

	All svMCI (*n* = 87)		PiB negative svMCI (*n* = 51)	
	B (SE)	*p*-Value	B (SE)	*p*-Value
Cognition				
Language	1.076 (0.699)	0.129	0.869 (0.693)	0.217
Visuospatial function	−0.277 (0.712)	0.699	0.949 (0.831)	0.260
Memory	1.873 (2.189)	0.395	2.792 (2.442)	0.259
Executive function	2.348 (3.396)	0.492	0.564 (3.912)	0.886
MMSE	0.419 (0.386)	0.281	0.301 (0.402)	0.457
CDR-SB	−0.034 (0.226)	0.880	−0.0665 (0.239)	0.782

Abbreviations: WMH—white matter hyperintensity; SE—standard error; MMSE—mini-mental status examination; CDR-SB—clinical dementia rating-sum of boxes. A linear mixed effects model was performed after adjusting for baseline age, sex, time, group, Pittsburgh compound B standardised uptake value ratio, and the interaction between the groups and time.

## Supplementary Materials

The following are available online at https://www.mdpi.com/2077-0383/9/8/2663/s1: Table S1, Correlation between delta WMH and longitudinal change of cortical thickness.
