# Supplementary material for: The Effects of Longitudinal White Matter Hyperintensity Change on Cognitive Decline and Cortical Thinning over Three Years
Source: J Clin Med. 2020 Aug 17;9(8):2663. doi: 10.3390/jcm9082663 (PMC7465642; doi:10.3390/jcm9082663)
Supplement: Supplementary file 1 [file jcm-09-02663-s001.pdf]

Table S1. Correlation between delta WMH and longitudinal change of cortical thickness.

	B value	***SE***	***p*-value**
Global thickness	-0.000014	0.000014	0.317
Frontal lobe	-0.000001	0.000016	0.141
Temporal lobe	-0.000001	0.000001	0.119
Parietal lobe	-0.000003	0.000014	0.842
Occipital lobe	-0.000010	0.000013	0.432
